# Infectious Sacroiliitis as a Rare Postpartum Complication: A Case Report

**DOI:** 10.7759/cureus.54621

**Published:** 2024-02-21

**Authors:** Renata Monteiro, Joana A Cabrera, Rui Salvador, Catarina Pereira, Marta Monteiro

**Affiliations:** 1 Internal Medicine, Centro Hospitalar de Vila Nova de Gaia/Espinho, Vila Nova de Gaia, PRT

**Keywords:** psoas muscle abscess, lumbar spine pain, post-partum, infectious sacroiliitis, pregnancy

## Abstract

Infectious sacroiliitis is a rare and challenging diagnosis. Sacroiliac joint changes related to pregnancy and puerperium can predispose to this condition. However, its clinical presentation can often mimic common causes of lower back pain and delay the diagnosis.

We report the case of a 31-year-old pregnant woman with twins who presented at 29 weeks of gestation with lower back and right buttock pain that radiated down to the thigh. Although initially interpreted as sciatica pain, the diagnosis of psoas hematoma complicated with infectious sacroiliitis was made. With this case, we aim to bring awareness to this condition in order to raise suspicion among clinicians for an earlier diagnosis.

## Introduction

Infectious sacroiliitis is a rare diagnosis that accounts for up to 10% of all cases of septic arthritis [1]. Predisposing factors include intravenous drug abuse, immune suppression, pregnancy, trauma, and an identifiable focus of infection elsewhere [2]. Infectious sacroiliitis can result from local contamination by contiguous infection or hematogenous spread from bacteriemia [1].

The clinical presentation varies greatly but the most frequent complaint is buttock and lower back pain that increases with walking. The sacroiliac joint is almost always tender at palpation and fever is usually present [2,3]. The flexion, abduction, and external rotation (FABER) test is a clinical pain provocation test that can be performed to identify problems with the hip and sacroiliac joint [2].

Its diagnosis can be difficult and frequently delayed because sacroiliitis presentation is similar to other common causes of lower back pain. Diagnosing sacroilliitis during pregnancy or postpartum is a particular challenge because pain in the lower back and buttocks is a common complaint during this period. In fact, during pregnancy, the release of relaxin increases the mobility of ligaments and the periarticular structures in the sacroiliac and pubic symphysis. This may cause a susceptibility to infection of the sacroiliac joint. Also, pregnancy-related immunosuppression contributes to an increased susceptibility [3-5].

Diagnosis should be confirmed by imaging methods with magnetic resonance imaging (MRI) being the most accurate imaging modality [4]. Radiographs are usually normal in the initial phases of septic sacroiliitis, and computed tomography (CT) performs poorly for identifying early changes in septic sacroiliitis, if cortical erosions, enlargement of the joint space, or inflammatory involvement of adjacent soft tissue are absent or minimal [3].

Staphylococcus aureus is the most common pathogen identified in infectious sacroiliitis, and streptococcus pyogenes is the second most common [1,6]. Initial therapy includes broad-spectrum intravenous antibiotics but there is no consensus regarding length of treatment [6].

Surgical treatment is indicated in cases of failure of antibiotic treatment or the presence of an abscess, bone destruction, septicemia, or neurological deficits and consists of debridement with or without joint arthrodesis [6,7]. The prognosis is usually good and improves with early diagnosis and adequate treatment before irreversible damage to the joint occurs [1].

## Case presentation

A 31-year-old female with a history of scoliosis and chronic back pain was pregnant with twins and admitted as an inpatient at 27 and 29 weeks of gestation, because of the symptomatic short cervix and increased risk of preterm delivery. At the second admission, she began complaining of back and right buttock pain radiating down the thigh that was interpreted as sciatica and she was prescribed tramadol and ibuprofen. She also started prophylaxis with 100 mg of aspirin and 40 mg of enoxaparin subcutaneously daily at discharge and until delivery. At 32 weeks of gestation, due to premature membrane rupture, eutocic delivery occurred, with no maternal complications.

At one week postpartum, she was evaluated at the emergency room complaining of increasing lumbar pain that radiated to the right buttock and thigh and was not responsive to the previously prescribed analgesic medication. Additionally, three days prior, she had developed a nonpruritic skin rash that started at the legs, but that was progressing to the arms and trunk. Palms and soles were not involved.

At admission, she was febrile (38ºC) and presented with elevated blood pressure (154/95mmHg) and normal heart rate (82 bpm). Neurologic examination was normal except for a limping gait. The rash was purpuric, maculopapular, palpable and non-blanching. A gynecological examination and ultrasound revealed no abnormalities. The laboratory findings revealed an elevated white blood cell count (16.58 x 10E3/µL), C-reactive protein (18.17 mg/dL), and erythrocyte sedimentation rate (>120 mm/Hr). The coagulation profile and protein electrophoresis were normal.

**Figure 1:**
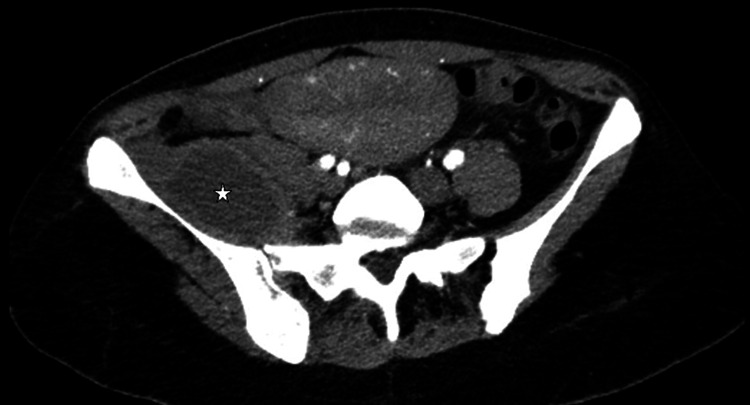
CT scan showing a psoas-infected hematoma in contiguity to the right sacroiliac joint The hematoma is marked with a star.

A CT was ordered and showed a right psoas hematoma with heterogeneous signal and septations that measured 72 x 45 x 120mm with anatomical proximity to the sacroiliac joint and possible compression of the S1 nerve root (Figure 1).

Assuming an infected spontaneous psoas hematoma, blood cultures were drawn, ceftriaxone and metronidazole were started, and percutaneous drainage was performed. Four days later, the antibiotic therapy was switched to flucloxacillin because a susceptible staphylococcus aureus was isolated from the blood cultures. The same bacteria was isolated from the pus drained. Endocarditis was excluded and she was afebrile 48 hours after starting antibiotics. Control blood cultures were negative.

Regarding the skin rash, blood tests were negative for autoimmunity and the skin biopsy revealed leukocytoclastic vasculitis, probably related to the ongoing infection. There was improvement with topical steroid therapy.

**Figure 2:**
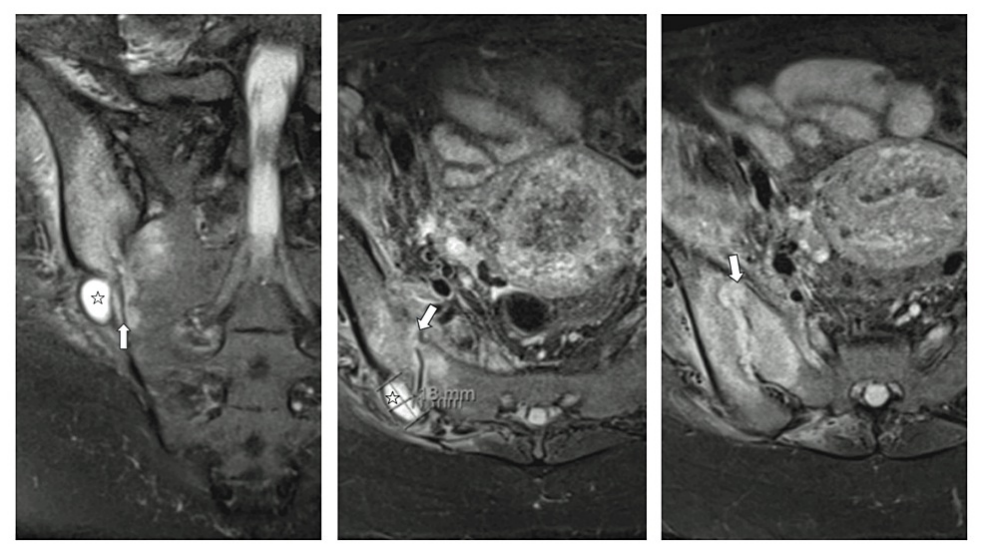
(Left to right) MRI of the sacroiliac joint showing an extensive right sacroiliitis and an adjacent abscess Inflammatory changes of the sacroiliac joint are marked with an arrow and the adjacent abscess is marked with a star.

After 15 days of antibiotics, she presented with persisting right buttock and thigh pain and an impaired gait with a positive FABER test. Another CT was ordered to assess the hematoma size and exclude osteomyelitis, which showed a significant decrease in hematoma size, now measuring 37 x 31 x 16 mm, but revealed an extensive infectious right sacroiliitis with severe bilateral bone involvement and widening of the joint space. Adjacent to the joint were two suspected abscesses later confirmed by MRI (Figure 2).

The patient was submitted to a surgical debridement without arthrodesis and completed a total of eight weeks of antibiotics with cefazolin in a hospital-at-home setting. After surgery, a physical therapy program was initiated, and the patient had a full functional recovery.

## Discussion

A spontaneous psoas hematoma and sacroiliac joint infection are rare entities and frequently missed. In the pregnancy and postpartum setting, although pelvic pain is frequent, a new onset of lombogluteal pain should alert clinicians for complications [1,3,5,8].

This case describes a spontaneous psoas hematoma with probable correlation with enoxaparin anticoagulation that got infected. The subsequent infection of the sacroiliac joint may have occurred by two different mechanisms (contiguity and hematogenous spread) because the patient presented with bacteriemia, and the hematoma was anterior to the joint. Bacteriemia may be related to the delivery.

The initial diagnostic delay was due to multifactorial. The patient had a history of scoliosis and chronic back pain and a clinical diagnosis of sciatica was made without an imagiological study. Since the psoas hematoma's proximity to the S1 nerve root explained most of the symptoms attributable to sciatica pain. Sciatic pain caused by infectious sacroiliitis has been described in literature due to irritating surrounding neural structures, such as the fifth lumbar nerve root (L5), and the first (S1) and second (S2) sacral nerve root, mimicking disk herniation. Therefore, clinical suspicion is important to pursue a diagnosis [9]. In addition, in the early stages of the infection, a radiograph and CT scan may not detect the sacroiliitis, which was a factor in this case [3].

Because an adjacent abscess was found in the MRI, surgical treatment was necessary. Surgical treatment should be considered in the presence of an abscess as a complement to the antibiotic treatment [6,7].

## Conclusions

Although rare, infectious sacroiliitis should be considered in postpartum patients who present with lumbar pain and fever. A delay in diagnosis increases the risk of joint and bone destruction which leads to a poor prognosis and increased morbidity. A thorough clinical exam combined with early MRI of the pelvis is the approach to achieving a timely diagnosis.
